# Monitoring Diagnostic Safety Risks in Emergency Departments: Protocol for a Machine Learning Study

**DOI:** 10.2196/24642

**Published:** 2021-06-14

**Authors:** Moein Enayati, Mustafa Sir, Xingyu Zhang, Sarah J Parker, Elizabeth Duffy, Hardeep Singh, Prashant Mahajan, Kalyan S Pasupathy

**Affiliations:** 1 Health Care Delivery Research Kern Center for the Science of Health Care Delivery Mayo Clinic Rochester, MN United States; 2 Amazon Care Seattle, WA United States; 3 Thomas E Starzl Transplantation Institute University of Pittsburgh Medical Center Pittsburgh, PA United States; 4 Department of Emergency Medicine University of Michigan Ann Arbor, MI United States; 5 Center for Innovations in Quality, Effectiveness and Safety Michael E DeBakey Veterans Affairs Medical Center Baylor College of Medicine Houston, TX United States

**Keywords:** diagnostic error, emergency department, machine learning, electronic health records, electronic triggers

## Abstract

**Background:**

Diagnostic decision making, especially in emergency departments, is a highly complex cognitive process that involves uncertainty and susceptibility to errors. A combination of factors, including patient factors (eg, history, behaviors, complexity, and comorbidity), provider-care team factors (eg, cognitive load and information gathering and synthesis), and system factors (eg, health information technology, crowding, shift-based work, and interruptions) may contribute to diagnostic errors. Using electronic triggers to identify records of patients with certain patterns of care, such as escalation of care, has been useful to screen for diagnostic errors. Once errors are identified, sophisticated data analytics and machine learning techniques can be applied to existing electronic health record (EHR) data sets to shed light on potential risk factors influencing diagnostic decision making.

**Objective:**

This study aims to identify variables associated with diagnostic errors in emergency departments using large-scale EHR data and machine learning techniques.

**Methods:**

This study plans to use trigger algorithms within EHR data repositories to generate a large data set of records that are labeled *trigger-positive* or *trigger-negative,* depending on whether they meet certain criteria. Samples from both data sets will be validated using medical record reviews, upon which we expect to find a higher number of diagnostic safety events in the trigger-positive subset. Machine learning will be used to evaluate relationships between certain patient factors, provider-care team factors, and system-level risk factors and diagnostic safety signals in the statistically matched groups of trigger-positive and trigger-negative charts.

**Results:**

This federally funded study was approved by the institutional review board of 2 academic medical centers with affiliated community hospitals. Trigger queries are being developed at both organizations, and sample cohorts will be labeled using the triggers. Machine learning techniques such as association rule mining, chi-square automated interaction detection, and classification and regression trees will be used to discover important variables that could be incorporated within future clinical decision support systems to help identify and reduce risks that contribute to diagnostic errors.

**Conclusions:**

The use of large EHR data sets and machine learning to investigate risk factors (related to the patient, provider-care team, and system-level) in the diagnostic process may help create future mechanisms for monitoring diagnostic safety.

**International Registered Report Identifier (IRRID):**

DERR1-10.2196/24642

## Introduction

### Background

Diagnostic decision making is a complex cognitive process involving uncertainty and susceptibility to errors. According to the National Academies of Science, Engineering, and Medicine, diagnostic error is defined as the “failure to establish an accurate and timely explanation of the patient’s health problem(s) or communicate that explanation to the patient” [1]. Approximately 30% of malpractice claims and more than 8% of adverse events in medicine are related to diagnostic errors [2], yet most are never reported [3]. Other researchers reported a 15% rate of diagnostic error in clinical medicine [2], with 5% of adults misdiagnosed annually in outpatient care [1,4] and about 15%-30% in the context of the emergency departments (EDs) [5].

The ED, in particular, is known as a *natural laboratory for the study of errors* [3], with a high prevalence of diagnostic errors [3]. Time-pressured decision making in a high-paced, high-volume, and chaotic ED environment increases the risk of erroneous diagnostic decisions [6]. Diagnostic errors are one of the most common types of errors in the ED [3]. Although precise error rates are lacking, they involve 65% of all closed malpractice claims [7]. A conservative estimate suggests that 1 out of every 10 diagnoses is subject to some level of error, and half of the errors cause harm or escalation of the health condition [1,8-10]. This results in approximately 7 million harmful errors out of the 139 million annual ED visits in the United States, making diagnostic safety a high priority for ED-related research [11].

Errors occurring in the ED often have multifactorial origins [12], and little is known about these factors [13]. In the absence of a unified taxonomy for contributing factors [14,15], the National Academies of Science, Engineering, and Medicine report [1] *Improving Diagnosis in Health Care* highlighted that the diagnostic process is not limited to the patient-provider interaction, and errors may result from the complex interplay of parameters related to patients (eg, health literacy, presenting symptoms, complexity, and behaviors), provider-care teams (eg, the cognitive load on providers and information gathering and synthesis), and systems (eg, health information technology, crowding, and interruptions).

There is an urgent need to design, implement, and develop novel methods to identify and reduce the risks related to the diagnostic process in complex ED microsystems. These methods should account for the dynamics of human-system interaction during the diagnostic process and address the inherent risks involved in these interactions. Efficiently screening large and ever-growing data sets, such as electronic health records (EHRs), can help identify cases with diagnostic errors and associated risk factors for mitigation strategies [16]. Data mining can be used to identify these factors and study their influence on diagnostic errors.

Data mining is a knowledge discovery method [17] that encompasses various algorithms to identify patterns and trends in large-scale data sets [18,19], such as EHRs. Previous research has shown the application of data mining techniques in the extraction of useful knowledge from large data sets in the fields of medicine and biology [20]. These algorithms use data to help scientists find input variables that have a significant relationship with the output variable. Most advancements in this type of analysis are achieved by incorporating techniques such as association rule mining (ARM), classification and regression trees (CART), and chi-square automated interaction detection (CHAID). ARM aims to extract frequent patterns, meaningful correlations, or causal structures within a data set [21] that satisfy the predefined minimum support and confidence from a given database [18]. This technique has been used in detecting disease co-occurrence, discovering adverse drug reactions, identifying risk factors for heart disease, and surveilling public health [22]. In contrast, decision trees are known to be effective in a variety of domains, with CART and CHAID being the two most popular decision tree techniques [17]. They are used to model the relationship between predictor variables and the outcome variable by recursive partitioning of large heterogeneous data sets into two or more homogeneous nodes [23].

### Objective

**Figure 1:**
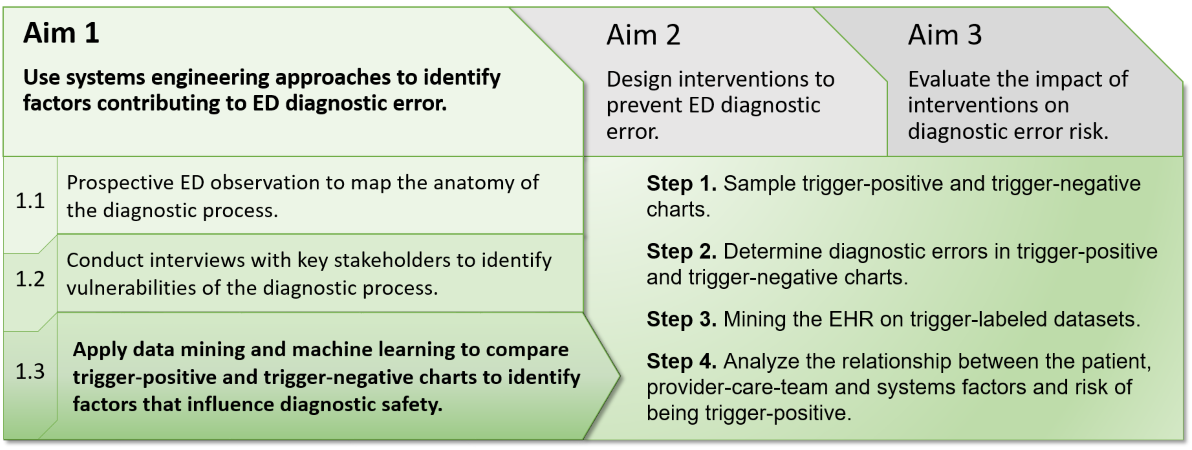
The 3 aims of the Agency for Healthcare Research and Quality-funded Improving Diagnosis in Emergency and Acute Care–Learning Laboratory project and the detailed steps of this study specifically focusing on aim 1.3 to identify patient-, provider-care team–, and system-level–factors affecting the risk for diagnostic error. ED: emergency department; EHR: electronic health record.

This protocol describes the application of data mining and machine learning techniques to understand diagnosis-associated risks in the ED and improve diagnostic safety. It focuses only on aim 1.3 of the recent *Improving Diagnosis in Emergency and Acute Care–Learning Laboratory* (IDEA-LL) grant awarded by the Agency for Healthcare Research and Quality (Figure 1). IDEA-LL is an actionable and patient-centered program for diagnostic safety surveillance and intervention based on the available data in EHRs. Diagnostic safety events will be identified through a review of EHR *triggers* related to events that are known to be associated with errors in the diagnosis process. After validation using EHR data, data mining and machine learning techniques will be utilized to compare an at-risk, trigger-positive sample with trigger-negative charts. This specific part of the grant will provide evidence-based guidance to identify factors with the highest prevalence among the trigger-positive cases and information that will be used in future projects (aim 2) to identify causal relationships and inform the design of decision support systems.

## Methods

### Setting

#### Population and Site Participation

Patient encounters from 4 EDs will be used in this study, including 2 from the Mayo Clinic system and 2 from the University of Michigan Health System. The institutional review boards from participating institutions approved the study protocol (19-009115, HUM00173662, 1696020-1). In this study, we will extract clinical data as part of data mining using triggers and compare them with nontriggered charts through the EHR systems at 4 EDs. Diversity of racial and ethnic backgrounds is represented in the 4 EDs by including all ages, races, ethnicities, and genders.

#### Sample Size Justifications and Power Calculation

We will estimate the sample size based on a power analysis with a two-tailed α=.05 and power of 85% for a predefined number of 10 independent variables that predict the diagnostic error yes or no outcome.

#### Sampling for Control

The control includes visits that do not meet the criteria for any trigger. We will include all ED visits in the two health systems for the entire study period (July 1, 2017-December 31, 2019). For each trigger-positive case, we will match a single trigger-negative encounter based on the availability of cases through a hierarchical procedure, which matches the encounters based on age group, gender, provider, the reason for visit, and the arrival date and time. This one-to-one matching of trigger-positive and trigger-negative cases will also help to eliminate the imbalance classes. To accommodate the potential heterogeneity across sites, the measurements will be reported as site-specific quartiles [24]. For patients with multiple ED or hospital visits during the study period, each record will be considered separately.

### Variables

#### Quantitative Variables

A variety of factors with potential influence on the diagnostic process will be extracted from both the EHR and other standalone databases at the 2 sites. Existing literature has provided information on factors related to patients, providers, and system-level parameters and the interactions of these parameters in the ED (eg, patient-per-provider ratio and patient length of stay), which can be explored further [8,13,24-29]. Several additional variables that can be extracted from the EHR will be under consideration. The number of unexpected ED visits could be associated with a higher risk of diagnostic error [8,25]. ED crowding is a complex issue related to both system-level and patient-level factors (complexity and acuity) [28] and is associated with an increased risk for patient safety, including treatment delays, reduced quality of care, and increased morbidity and mortality [24]. Prolonged ED length of stay correlates with increased patient mortality [27]. High workloads, lack of control, and communication failures may lead to patient safety risks [29]. Iordache et al [30] showed that direct and indirect care time together are significant discriminators between EDs because of the differences between their patient care profiles and unit characteristics. Prescribing error rates are shown to significantly increase if physicians are interrupted or are multitasking [31]. Textbox 1 provides a general overview of these 3 categories.

**The three main categories of risk factors to be explored in this study.**

**Patient Factors**

- Demographics
- Emergency department (ED) visit history
- Complexity and comorbidity

**Provider-Care Team Factors**

- Shift schedule and workload
- Patient-per-provider ratio
- Interruptions and trauma

**System Factors**

- ED boarding
- Emergency severity index triage distribution
- Total direct and indirect care time

#### Qualitative Variables

Qualitative analysis of the ED environments, such as the cognitive load on individual providers, is outside the focus of this study but is under exploration in another aim of this grant. However, we will study factors such as patient acuity, patient volume, waiting times, number of ED providers per shift, and number of boarded ED patients (admitted to inpatient unit but still in ED because of lack of inpatient space) in the system as proxies for some of the qualitative factors such as the cognitive load.

### Data Quality and Safety Monitoring

We will evaluate compliance with the study methodology, quality of available data, patient protection, and adherence to Good Clinical Practice guidelines. We anticipate differences in terms of practice among the sites that may impact data quality. To ensure that the practice differences are accounted for, we recruited clinicians at each site to determine important differences and help customize the trigger algorithms. In addition, data consistency and completeness will be audited using data queries designed in accordance with standard techniques. Meanwhile, potential inconsistencies and missing values will be identified during the clinician chart reviews to design and apply adequate data imputations.

The EHR systems at both sites are the same, and we use standard measurements through health systems, with some minor customizations. These standard measurements will work as proxies to better identify conditions that may result in an error, so we do not anticipate that differences in practices across sites will affect data quality. Inconsistent data will be used to examine and enhance the validity of the defined measures (triggers) and assess their performance characteristics as predictive values. Data will be screened for missing values, and most of the missing data elements will be replaced by the closest available proxy in the EHR. As both sites use Epic as their official EHR system, care providers and Epic specialists at both sites will be engaged in this discussion to accommodate potential deployment variations. In the rare event of technical problems, remaining missingness will be adequately handled by missing data techniques such as data imputation or maximum likelihood estimations [5]. Incorrect data entries in the Epic system are very difficult to identify because of the lack of other reference ground truths. However, we will perform a data cleaning procedure to ensure the meaningfulness of temporal information, and incorrect timings (such as negative values for the length of stay) will be replaced by approximations based on the follow-up events. We will also report the missing data rate and dropout rates.

### Conduct of the Study

#### Overview

This study aims to identify the factors that may interfere with the diagnostic process in the ED that potentially lead to missed, delayed, or incorrect diagnoses. On the basis of our previous work, we will use a series of electronic triggers (triggers) to identify ED encounters with potential diagnostic errors from the EHR database. Each trigger has a predefined set of inclusion and exclusion criteria implemented in Structured Query Language (SQL) and configured for specifications of each site in the study. Each site currently uses an Epic EHR system with minimal variations owing to its specific needs. These differences will be identified and accounted for after the focus interviews with the providers. The Epic specialists at both sites are in constant communication with the research team to apply the most accurate mapping of the factors and parameters between the 2 sites. The protocol is thought to be generalizable to other EHR systems, as we emphasize the analysis of common ED concepts rather than database variables specific to Epic. All SQL queries can be modified to match other systems (eg, Cerner) by matching concepts and keywords. Table 1 provides an initial list of 6 EHR-based triggers proposed in IDEA-LL after reviewing the literature on current triggers, surveying medical directors, and using a Delphi consensus process [32]. We will start our study with the first 3 triggers, including the unscheduled visit within 10 days resulting in admission, care escalation to intensive care unit within 24 hours, or death in the ED or within 24 hours of ED departure time. If for any reason, one or more of the triggers do not perform well, the triggers from the backup set will be used.

**Table 1:** Proposed electronic trigger algorithms for identifying diagnostic errors.

Trigger algorithm			Description
**Initial set of triggers**			
	Trigger 1: unscheduled return	Unscheduled return visit with admission within 7 to 10 days from the index ED^a^ visit.	
	Trigger 2: care escalation	Care escalation from the inpatient unit to the ICU^b^ within 6, 12, or 24 hours with ED attribution.	
	Trigger 3: death	All deaths in the ED or within 24 hours of admission—exclusive of palliative care.	
**Backup set of triggers**			
	Trigger 4: change of service	A proxy for the discrepancy in diagnosis may be the change of service in 48 hours (admitted medical, changed to surgical).	
	Trigger 5: nonadmitted returns	Return visits not resulting in admission with new interventions (eg, diagnostic test that was abnormal, new medication).	
	Trigger 6: high-risk conditions	New diagnosis or symptom-disease dyads (eg dizziness-stroke [33] and abdominal pain-appendicitis [34]).	

^a^ED: emergency department.

^b^ICU: intensive care unit.

We will perform a retrospective review of a selected sample of both trigger-positive and trigger-negative medical records to identify the presence or absence of diagnostic errors using the Revised Safer Dx, a validated instrument for categorizing the presence of diagnostic errors [35]. We will then compare these 2 large cohorts to evaluate associations of potential contributing factors with trigger-positive cohorts using sophisticated data mining techniques. To conduct this study, we will accomplish the following 4 steps.

#### Step 1: Sampling of Trigger-Positive and Trigger-Negative Charts

This step will apply the EHR-based trigger algorithms (Table 1) to the EHR data repositories that include ED encounters at the 2 sites, creating large data sets of statistically matched groups (for demographics and medical comorbidity or severity) for trigger-positive and trigger-negative (control) charts [36]. The inclusion and exclusion criteria for individual triggers will be refined after an iterative review of random samples of charts. We anticipate using 3 triggers that have the best predictive value for diagnostic errors.

#### Step 2: Determination of Diagnostic Errors in Trigger-Positive and Trigger-Negative Charts

This step aims to determine whether diagnostic errors are statistically more likely to appear in trigger-positive charts. We will investigate the presence of diagnostic error through manual review of a sample of trigger-positive and trigger-negative charts using the Revised Safer Dx instrument [35]. For each trigger, we will calculate the odds ratio of a diagnostic error for both trigger-positive and trigger-negative groups and perform appropriate hypothesis tests to decide if trigger-positive charts are more likely to result in a diagnostic error.

#### Step 3: Mining the EHR on the Extracted Trigger-Labeled Data Sets

The triggers for which we fail to reject the null hypothesis will be selected for further analysis. We will use EHR-based queries to automatically label ED patient charts as trigger-positive or negative. This data set also includes several potential predictive factors extracted from the EHR, in addition to the trigger labels. We will also consider factors previously underinvestigated but recognized anecdotally, as listed in Textbox 1, for the 3 categories of patient-related, provider-related, and system-related factors. Some factors can be directly extracted from the EHR database, such as arrival time, ED arrival rate, emergency severity index triage algorithm distribution, that is, severity of ED workload. We will ensure equal distribution of trigger-positive and trigger-negative data sets by the site. We can overlay these 3 sets of factors to mirror each patient’s journey during the ED stay.

**Figure 2:**
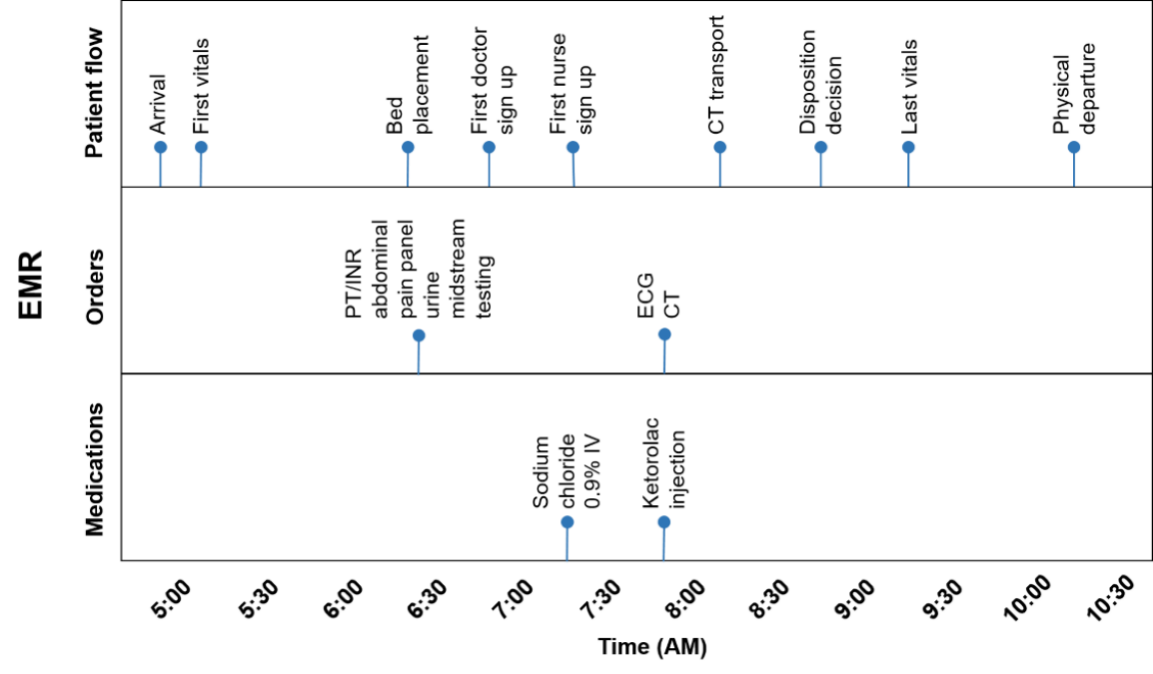
Example of emergency department visit information flow. CT: computerized tomography; ECG: electrocardiogram; EMR: Electronic medical record; IV: intravenous; PT/INR: Prothrombin time and international normalized ratio.

**Figure 3:**
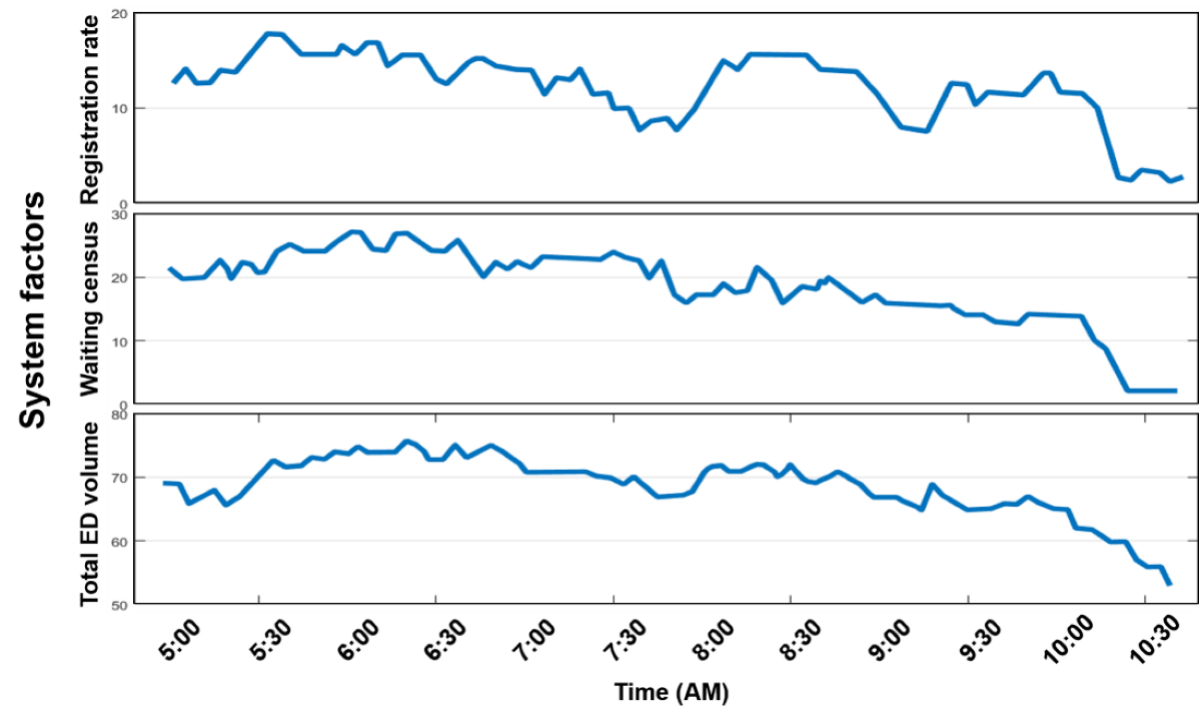
Emergency department context impacting the diagnostic process. ED: emergency department.

For example, consider a hypothetical 70-year-old patient presenting to the ED with left flank pain. The partial information flow associated with this patient’s ED visit is illustrated in Figure 2, where patient-related diagnostic events are demonstrated by time. Figure 3 demonstrates important contextual information such as ED volume, waiting room census, and patient arrival rate, all of which can impact or delay the diagnostic process. The 2 figures provide a side-by-side example of variations in patient- and system-level factors that could influence the prevalence of diagnostic error. Such associations among these factors can only be identified by considering all possible influencers in the ED environment.

#### Step 4: Analysis of the Relationship Among Patient, Provider-Care Team, and System Factors and the Risk of Being Trigger-Positive

##### Overview

This step aims to analyze the trigger-labeled data sets obtained from the 2 institutions using different machine learning and data mining techniques. Data mining is a prevalent and effective technique for extracting nontrivial, implicit, previously unknown, and potentially useful knowledge from large data sources [37]. Discovering significant information related to disease diagnosis from medical databases is possible by applying techniques such as ARM [22], CHAID, and CART.

##### Association Rule Mining

ARM has been successfully applied in various medical contexts, from the discovery of adverse drug reactions to the identification of risk factors for heart disease [22]. ARM is one of the most significant unsupervised methods for pattern recognition [37], which explores frequently occurring patterns to find hidden associations between different factors. ARM will estimate the likelihood of trigger-positive risk through different factor combinations. Its predictive a priori model combines confidence and support into a single measure of predictive accuracy and discovers the best associations among the factors in large data sets [38]. Rules extracted by this method are usually represented in IF-THEN form, which makes it easier for medical experts to interpret and comprehend medical analysis [22].

##### Chi-square Automated Interaction Detection

CHAID is a decision tree algorithm that determines splitting based on statistical tests and has been used to model the relationship between the predictor variables and the outcome variable in many medical applications, such as identifying factors influencing inpatient mortality [17]. In our study, CHAID will help answer the question “Which combination of factors leads to higher trigger-positive risk and therefore a higher relative risk of diagnostic error?” CHAID splits the target into two or more categories using an exploratory analysis of the relationship between a dependent factor and several predictor factors [39]. To see if splitting the sample based on these predictors leads to statistically significant discrimination in the dependent measure (trigger labels), various independent factors will be evaluated using the chi-square test [40]. Despite regression, CHAID is capable of illustrating variable clusters through an iterative process. Adjusted *P* value measures are used to determine the best value of the partition or the best split, and splitting on a larger chi-square statistic indicates a more significant partition.

##### Classification and Regression Trees

CART analysis is another tree-based nonparametric data mining technique frequently used in medical diagnosis studies [23]. It has been widely used in the literature for both classification and interpretation tasks, such as identifying important predictive factors for persistent shoulder pain [41], ranking the risk factors for *Schistosoma mansoni* reinfection [42], and analyzing the risk factors of hypertension [43]. CART divides a large heterogeneous data set into smaller, more homogeneous nodes by employing recursive partitioning based on a target variable [23]. The significance of these decision rules is the definition of subgroups of patients and the most relevant interactions between them [44].

## Results

The entire study cohort is well specified and labeled by trigger scripts, and the data are undergoing cleaning and preparation for subsequent steps. Following the completion of this study, we expect to characterize common factors associated with both trigger-positive and trigger-negative charts by applying multiple machine learning-based factor analysis techniques. These algorithms explore different combinations of factors within all trigger-positive and trigger-negative cases to identify meaningful interactions of risk factors concerning each trigger. We have already provided a list of potentially important EHR-based factors for the triggers based on our literature review and previous experiences in practice. As listed in Textbox 2, some of these factors are common among all 3 triggers, and some are specific to a trigger based on the definition. We will also expand and explore a list of previously underinvestigated but recognized anecdotally as potential patient-, provider-care team–, and system-related factors that could be approximated by these variables (eg, estimating the effect of the cognitive burden by system and provider variables such as ED crowding).

**List of potential electronic health record–based predictor variables extracted from previous studies.**

**Factors Across All Triggers**

- Demographics (eg, age and sex)
- Emergency department (ED) encounter (date and time [D/T])
- Chief complaints
- Clinical impression (tenth revision of the International Statistical Classification of Diseases and Related Health Problems [ICD10])
- Final ED diagnosis (ICD10)
- Hospital admission diagnosis
- ED length of stay
- Number of lab tests

**Trigger 1: Specific Factors**

- Previous ED departure (D/T)
- Previous ED disposition
- Return ED arrival (D/T)
- Return ED disposition
- Return ED type
- Return ED chief complaints
- Return ED clinical impression
- Return ED diagnosis (ICD10)
- Return hospital diagnosis

**Trigger 2: Specific Factors**

- ED discharge (D/T)
- Hospital disposition
- Next unit
- Intensive care unit (ICU) unit
- Time-to-ICU (hours)

**Trigger 3: Specific Factors**

- ED discharge (D/T)
- ED disposition
- ED departure (D/T)
- ED unit
- Discharge-to-death (hours)
- Hospital disposition

**Figure 4:**
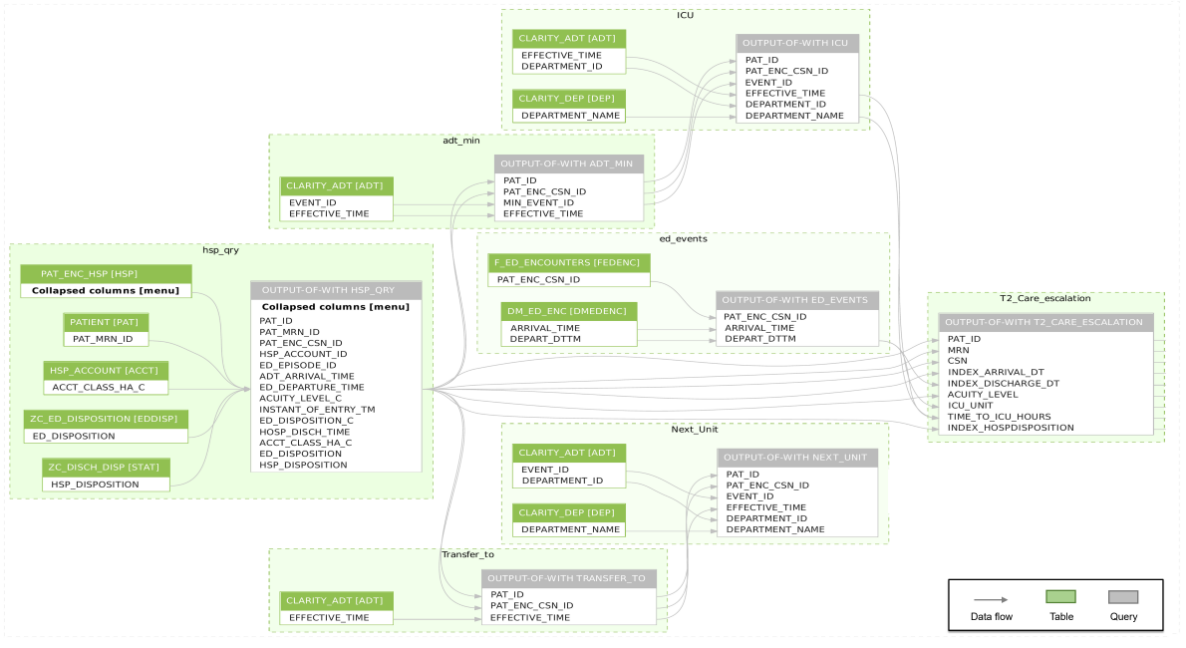
Emergency department patients’ evaluation against each trigger is done upon executing these queries on each electronic health record database. This figure is an example relational database schema on how relevant electronic health record–based predictive factors are being invoked from multiple database tables to build up a record of escalations in the care condition, denoted by trigger-2. Abbreviations in the figure represent arbitrary variable names as examples.

We have also updated the SQL queries of all 3 triggers to be compatible with the EHR databases at both sites. ED patients’ evaluations against each trigger are performed upon the execution of these queries on each EHR database. We reinforced multiple inclusion and exclusion criteria to the queries to ensure that test data or irrelevant information do not affect our selections. All queries share common information related to the initial encounter, followed by specific factors collected for each trigger separately. Figure 4 shows an example relational database schema on how different factors are being invoked and matched from multiple database tables to identify encounters with an escalation in the care condition, denoted by trigger 2.

The outcomes of this project include an improved understanding of the risk factors contributing to diagnostic error in the ED. The data could be used to inform EHR-based decision support systems for better prediction of the risk of diagnostic error in the ED. The first exploratory results of the project are expected to be submitted for publication by mid-2021.

## Discussion

### Relevance

A strategy to address diagnostic error is the better use of health information technology and EHR data [45]. This project expands upon previous work on diagnostic errors by investigating risk factors through data mining techniques applied to EHR data. In particular, we aim to generate information relevant to the future design of a dynamic ED-based decision support system to enhance the quality of emergency care through large-scale analysis of EHR records. As a result of using unbiased machine learning techniques, we will find previously unexplored associations. These novel associations will help future investigations into causal risk factors for diagnostic errors.

Building on this in the future (Figure 1, aim 3), we will use machine learning approaches with patient and frontline provider factors to develop a real-time, dynamic, trigger-based EHR diagnostic error risk prediction tool. This will inform clinicians of potential risks based on the patients’ EHR records that could be preventable or addressed through appropriate intervention.

In contrast, interfacing with other systems such as health information exchanges may improve the completeness of clinical information on patients and may help improve the predictive value of triggers (such as when a return visit was in a nearby ED that participated in a health information exchange vs not). We will be open to exploring all kinds of such interactions, and if we see *signals* regarding medications or other systematic alerts, we will study them further by obtaining clinician’s insights for face validity and accuracy. Although we are not specifically focused on drug or medication-related events, if they contribute to the diagnostic process and breakdowns, they will be included as covariates.

### Limitations and Strengths

We anticipate some difficulties and potential limitations to our forthcoming project. First, although we have based our analysis on the patients’ medical records, it is notable that this information is not always available at all sites. Our method potentially cannot be extended to EDs that do not have an integrated EHR system, as different EDs might use incompatible EHR systems to ours, which at least requires an adaptation phase. We intentionally chose the community and academic sites to learn more about the potential difference and have recruited clinicians at each site to determine important differences and help customize the trigger algorithms. We also believe that by incorporating commonly accepted and standard measurements in this study, any future translation to other health systems would be possible by a simple mapping to the database elements of the target EHR system.

Second, our approach to investigate the risk of diagnostic error for ED patients with care escalation or death within a certain period from ED discharge will not include many events, including those that occur within the same ED encounter, possible events in other hospitals, and flag records that meet trigger criteria but are not associated with diagnostic error. We will attempt to update our data exploration approach to reduce the rate of such false-positive triggers. Conversely, a strength of our study is that the simultaneous investigation of 2 separate health systems with both adult and pediatric EDs reduces the chance of biased conclusions. These limitations are balanced by several unique strengths of this study to help identify potentially associated contributory factors of diagnostic error in the ED.

### Conclusions

The use of sophisticated data mining techniques to compare trigger-positive and trigger-negative records will enrich the list of risk factors that lead to diagnostic errors. To the best of our knowledge, this is the first application of exploratory decision tree techniques such as CART and CHAID to determine the relative importance of associated predictors of diagnostic error. Such techniques will help identify risk factors of diagnostic error using EHR data and inform the development of future dynamic ED-based decision support systems for monitoring and improving diagnostic safety.

## Abbreviations

ARM: association rule mining
CART: classification and regression tree
CHAID: chi-square automated interaction detection
ED: emergency department
EHR: electronic health record
IDEA-LL: Improving Diagnosis in Emergency and Acute Care–Learning Laboratory
SQL: Structured Query Language

## References

[1] National Academies of Sciences, Engineering, and Medicine, Institute of Medicine, Board on Health Care Services, Committee on Diagnostic Error in Health Care (2015). Improving Diagnosis in Health Care.

[2] van den Berge K, Mamede S (2013). Cognitive diagnostic error in internal medicine. Eur J Intern Med.

[3] Okafor N, Payne VL, Chathampally Y, Miller S, Doshi P, Singh H (2016). Using voluntary reports from physicians to learn from diagnostic errors in emergency medicine. Emerg Med J.

[4] Makridakis S, Kirkham R, Wakefield A, Papadaki M, Kirkham J, Long L (2019). Forecasting, uncertainty and risk; perspectives on clinical decision-making in preventive and curative medicine. Int J Forecast.

[5] Hautz SC, Schuler L, Kämmer JE, Schauber SK, Ricklin ME, Sauter TC, Maier V, Birrenbach T, Exadaktylos A, Hautz WE (2016). Factors predicting a change in diagnosis in patients hospitalised through the emergency room: a prospective observational study. BMJ Open.

[6] Roosan D, Law AV, Karim M, Roosan M (2019). Improving team-based decision making using data analytics and informatics: protocol for a collaborative decision support design. JMIR Res Protoc.

[7] Kachalia A, Gandhi TK, Puopolo AL, Yoon C, Thomas EJ, Griffey R, Brennan TA, Studdert DM (2007). Missed and delayed diagnoses in the emergency department: a study of closed malpractice claims from 4 liability insurers. Ann Emerg Med.

[8] Singh H, Meyer AN, Thomas EJ (2014). The frequency of diagnostic errors in outpatient care: estimations from three large observational studies involving US adult populations. BMJ Qual Saf.

[9] Graber ML (2013). The incidence of diagnostic error in medicine. BMJ Qual Saf.

[10] Croskerry P, Sinclair D (2001). Emergency medicine: a practice prone to error?. Can J Emerg Med.

[11] (2017). Emergency department visits. Centers for Disease Control and Prevention.

[12] Zavala AM, Day GE, Plummer D, Bamford-Wade A (2018). Decision-making under pressure: medical errors in uncertain and dynamic environments. Aust Health Review.

[13] Hautz WE, Kämmer JE, Hautz SC, Sauter TC, Zwaan L, Exadaktylos AK, Birrenbach T, Maier V, Müller M, Schauber SK (2019). Diagnostic error increases mortality and length of hospital stay in patients presenting through the emergency room. Scand J Trauma Resusc Emerg Med.

[14] Zwaan L, Singh H (2015). The challenges in defining and measuring diagnostic error. Diagnosis (Berl).

[15] Newman-Toker DE (2014). A unified conceptual model for diagnostic errors: underdiagnosis, overdiagnosis, and misdiagnosis. Diagnosis (Berl).

[16] Murphy DR, Meyer AN, Sittig DF, Meeks DW, Thomas EJ, Singh H (2019). Application of electronic trigger tools to identify targets for improving diagnostic safety. BMJ Qual Saf.

[17] Chae YM, Kim HS, Tark KC, Park HJ, Ho SH (2003). Analysis of healthcare quality indicator using data mining and decision support system. Expert Syst Appl.

[18] Son LH, Chiclana F, Kumar R, Mittal M, Khari M, Chatterjee JM, Baik SW (2018). ARM–AMO: An efficient association rule mining algorithm based on animal migration optimization. Knowl Based Syst.

[19] Lin J, Gan W, Fournier-Viger P, Hong T, Tseng V (2016). Fast algorithms for mining high-utility itemsets with various discount strategies. Adv Eng Inform.

[20] Lahiri D, Dubey S, Ardila A, Sanyal D, Ray BK (2020). Determinants of aphasia recovery: exploratory decision tree analysis. Lang Cogn Neurosci.

[21] Ratner B (2012). Statistical and Machine-Learning Data Mining: Techniques for Better Predictive Modeling and Analysis of Big Data.

[22] Borah A, Nath B (2018). Identifying risk factors for adverse diseases using dynamic rare association rule mining. Expert Syst Appl.

[23] Bishop JC, Rinn AN (2019). The potential of misdiagnosis of high IQ youth by practicing mental health professionals: a mixed methods study. High Abil Stud.

[24] Epstein SK, Huckins DS, Liu SW, Pallin DJ, Sullivan AF, Lipton RI, Camargo CA (2012). Emergency department crowding and risk of preventable medical errors. Intern Emerg Med.

[25] Fontil V, Khoong EC, Hoskote M, Radcliffe K, Ratanawongsa N, Lyles CR, Sarkar U (2019). Evaluation of a health information technology-enabled collective intelligence platform to improve diagnosis in primary care and urgent care settings: protocol for a pragmatic randomized controlled trial. JMIR Res Protoc.

[26] Khajehali N, Alizadeh S (2017). Extract critical factors affecting the length of hospital stay of pneumonia patient by data mining (case study: an Iranian hospital). Artif Intell Med.

[27] Pryce A, Unwin M, Kinsman L, McCann D (2021). Delayed flow is a risk to patient safety: a mixed method analysis of emergency department patient flow. Int Emerg Nurs.

[28] Morley C, Unwin M, Peterson GM, Stankovich J, Kinsman L (2018). Emergency department crowding: a systematic review of causes, consequences and solutions. PLoS One.

[29] Källberg A, Ehrenberg A, Florin J, Östergren J, Göransson KE (2017). Physicians' and nurses' perceptions of patient safety risks in the emergency department. Int Emerg Nurs.

[30] Iordache S, Elseviers M, De Cock R, Van Rompaey B (2020). Development and validation of an assessment tool for nursing workload in emergency departments. J Clin Nurs.

[31] Westbrook JI, Raban MZ, Walter SR, Douglas H (2018). Task errors by emergency physicians are associated with interruptions, multitasking, fatigue and working memory capacity: a prospective, direct observation study. BMJ Qual Saf.

[32] Mahajan P, Pai C, Cosby KS, Mollen CJ, Shaw KN, Chamberlain JM, El-Kareh R, Ruddy RM, Alpern ER, Epstein HM, Giardina TD, Graber ML, Medford-Davis LN, Medlin RP, Upadhyay DK, Parker SJ, Singh H (2020). Identifying trigger concepts to screen emergency department visits for diagnostic errors. Diagnosis (Berl).

[33] Newman-Toker DE, Moy E, Valente E, Coffey R, Hines AL (2014). Missed diagnosis of stroke in the emergency department: a cross-sectional analysis of a large population-based sample. Diagnosis (Berl).

[34] Mahajan P, Basu T, Pai C, Singh H, Petersen N, Bellolio MF, Gadepalli SK, Kamdar NS (2020). Factors associated with potentially missed diagnosis of appendicitis in the emergency department. JAMA Netw Open.

[35] Singh H, Khanna A, Spitzmueller C, Meyer AN (2019). Recommendations for using the Revised Safer Dx Instrument to help measure and improve diagnostic safety. Diagnosis (Berl).

[36] Lee M (2016). Matching, Regression Discontinuity, Difference in Differences, and Beyond.

[37] Telikani A, Gandomi AH, Shahbahrami A (2020). A survey of evolutionary computation for association rule mining. Inf Sci.

[38] Witten IH, Frank E (1999). Data Mining: Practical Machine Learning Tools and Techniques with Java Implementations.

[39] Kass GV (1980). An exploratory technique for investigating large quantities of categorical data. Appl Stat.

[40] Lin C, Fan C (2019). Evaluation of CART, CHAID, and QUEST algorithms: a case study of construction defects in Taiwan. J Asian Archit Build Eng.

[41] Chester R, Khondoker M, Shepstone L, Lewis JS, Jerosch-Herold C (2019). Self-efficacy and risk of persistent shoulder pain: results of a Classification and Regression Tree (CART) analysis. Br J Sports Med.

[42] Gazzinelli A, Oliveira-Prado R, Matoso LF, Veloso BM, Andrade G, Kloos H, Bethony JM, Assunção RM, Correa-Oliveira R (2017). Schistosoma mansoni reinfection: analysis of risk factors by classification and regression tree (CART) modeling. PLoS One.

[43] Fernanda JW, Anuraga G, Fahmi MA (2018). Risk factor analysis of hypertension with logistic regression and Classification and Regression Tree (CART). J Phys: Conf Ser, Vol 1217, Proceedings of the 8th International Seminar on New Paradigm and Innovation on Natural Science and Its Application.

[44] Phillips-Wren G, Sharkey P, Dy SM (2008). Mining lung cancer patient data to assess healthcare resource utilization. Expert Syst Appl.

[45] Ball JR, Balogh E (2016). Improving Diagnosis in Health Care: highlights of a report from the national academies of sciences, engineering, and medicine. Ann Intern Med.

